# Impact of yoga and exercises on polycystic ovarian syndrome risk among adolescent schoolgirls in South India

**DOI:** 10.1002/hsr2.212

**Published:** 2020-12-04

**Authors:** Valarmathi Selvaraj, Jain Vanitha, Fabiola M. Dhanaraj, Prema Sekar, Anitha Rajendra Babu

**Affiliations:** ^1^ Nursing Meenakshi Academy of Higher Education and Research Chennai India; ^2^ Meenakshi College of Nursing Chennai India; ^3^ Arulmigu Meenakshi College of Nursing Kancheepuram India; ^4^ Annai Veilankanni's College of Nursing Chennai India; ^5^ Rajalakshmi College of Nursing Chennai India

**Keywords:** adolescent girls, exercise, lifestyle modifications, polycystic ovarian syndrome, risk assessment, school girls, yoga

## Abstract

**Background:**

To identify the adolescent school girls with risk for polycystic ovarian syndrome (PCOS), assess their risk status, and evaluate the impact of lifestyle modifications on PCOS risk reduction.

**Methods:**

An experimental research was conducted among adolescent girls belonging to two Government run schools in Tiruvallur district of Tamil Nadu state, India, from 6 June to 9 December 2016. A standard risk assessment questionnaire was adopted for risk assessment after making few modifications (Cronbach alpha 0.86). The experimental group received lifestyle modifications (yoga for two months and walking exercise for two months), with no such intervention provided for the control group. The impact of these interventions was assessed in terms of risk minimization and a *P* value less than .05 was considered statistically significant.

**Results:**

A total of 204 (control—102; experimental—102) girls with statistically insignificant difference in demographic features were studied. During the pretest, 85.2% (n = 87) in the experimental group and 83.3% (n = 85) the controls had “moderate risk” for PCOS. Girls with “high risk” level of PCOS were 14.8% (n = 15) and 15.7% (n = 17) in the experimental group and the control group, respectively. In posttest‐1 (after yoga sessions) risk assessment, 71.6% had “moderate risk,” 5.9% had “high risk” in the experimental group, whereas 87.3% had “moderate risk” and 12.7% had “high risk” in the control group. In posttest‐2 (after exercise sessions) risk assessment, 48% had “moderate risk” and 0% had high risk in the experimental group, whereas 88.2% were “moderate risk” and 11.8% were “high risk” in the control group. Repeated measure ANOVA with Greenhouse‐Geisser correction showed mean risk reduction score statistically significant between pretest and post‐test (33.38 ± 7.28 vs 22.75 ± 12.09, respectively mean difference is 10.63: *F* = 236.12 *P* < .001), suggesting a positive correlation with the intervention.

**Conclusions:**

Yoga and exercise were beneficial in minimizing PCOS risk, as reflected in the risk assessment score. More such interventions, covering different schools, could provide larger health benefits.

## 1 BACKGROUND

Polycystic ovarian syndrome (PCOS) is a disorder affecting females of reproductive age, characterized by amenorrhea, obesity, and hirsutism, and is associated with enlarged polycystic ovaries.^1^ The signs and symptoms include menstrual irregularity, obesity, and excessive hair growth on the face and chest.^2^, ^3^ Psychological disorder, including depression, anxiety, bipolar disorders, stress, sleep apnea, can worsen the Quality of Life (QoL).^4^ The major complications associated with PCOS are infertility, diabetes, cardiovascular diseases, dyslipidemia, hypertension, glucose intolerance, and metabolic syndrome.^5^, ^6^, ^7^ There are several medications available for treating PCOS, with varying success levels and are associated with drug‐related problems. Evidence shows lifestyle modifications as a viable first‐line effective means for preventing PCOS.^8^, ^9^, ^10^, ^11^, ^12^ Even a small change in lifestyle helps to decrease the risk for PCOS.^13^ It is evident that there are no medications that can completely cure PCOS.^14^ It has been well documented that lifestyle modifications, such as proper diet,^15^ yoga, and exercise,^16^ help in decreasing the symptoms and severity of the disease.

In India, the prevalence of PCOS is highly variable, ranging from 2.2% to 26%.^17^ The prevalence of PCOS in South Indian states like Andhra Pradesh is 9.13%,^18^ within the same state Nellore district has a prevalence of 15.4%.^19^ Telangana, a neighboring state in South India, has a prevalence of about 20%.^20^ In Bangalore city of Karnataka state, PCOS is gradually increasing and is becoming an epidemic.^21^ PCOS prevalence among the age group between 18 and 25 years is 3.7% in Lucknow, a city in North India,^22^ 46.8% in New Delhi, the capital city of India, and 26.4% in Kerala, a south Indian state.^23^ Furthermore, the prevalence rate of PCOS among medical undergraduate girls in Pondicherry was 12.18%, even though they had basic knowledge about the reproductive system and PCOS,^24^ and 9.8% in Thiruvananthapuram,^25^ both these cities are in South India. These observations show a rising prevalence of PCOS in India.

On a daily observation, one can note a drastic change in the lifestyle of Indian population, which is more often linked to the globalization in India during the past three decades. During ancient days, Indian women were exposed to household physical work such as pulling water from the well, working in the field, washing clothes with hands, making decorative arts in their homes (Rangoli), grinding flour using Indian grinding machine (Ammi Kal), and so on. All these household works are likely to be linked to an improved endocrine function. Due to the advancement in technology, these natural lifestyle exercises are being replaced with modern devices and technologies. Adolescent girls spend their time in watching television, playing video games, smartphone, and other gadgets. Thus, the scope for physical exercises is limited. Lifestyle modifications such as yoga and exercise are associated with less risk, least cost, does not require a visit to a health club or gymnasium, and do not cause any negative effects on the reproductive system of adolescent girls. Thus, it is worth considering lifestyle modifications as a promising method for reducing the risk of PCOS.

Schools can also serve as a platform for providing lifestyle education to students. School children are at the earliest stage of life hood, and hence are open to accept changes, so they can be molded easily by teachers and parents. The school‐going adolescent girls are proven to be attentive and are willing to listen. Schools could also involve the teachers who could be very much valuable in identification, motivation, and training the students. Moreover, children from Government run schools and their parents may not be aware of the risk of PCOS due to their poor socioeconomic background, less exposure to the internet and smartphone devices, and low literacy level. Thus, schools can be considered as an ideal setting to provide education on lifestyle changes and related awareness in an attempt to reduce PCOS risk.

Risk assessment appears to be the most useful method for identifying the condition at the earliest stage and encourage the adolescents to seek for interventions to PCOS development as there is no complete cure for it.^26^ There have been many types of research studies done in this area, but no specific research has been conducted to deal with the risk assessment and impact of lifestyle modifications on PCOS among school going adolescent girls. All these scenarios provoked the researcher to undertake this study with the objectives of identifying the adolescent girls with risk for PCOS, and to evaluate the impact of yoga and exercise on PCOS risk among the adolescent girls.

## 2 METHODS

### 2.1 Research design

In this research, a “true experimental research” design was adopted, and the test group received interventions with no intervention provided for the experimental group.

### 2.2 Study population

Adolescent girls studying in Government Girls Higher Secondary School in Keelmanambedu and Government Girls Higher Secondary School, Poonamallee in Thiruvallur district of Tamil Nadu, India. The sample comprised of adolescent girls studying in 10th to 12th grades.

### 2.3 Ethical consideration

Prior to the study, ethical approval was obtained from the Billroth Hospital Ethical Review Board, Chennai, Tamil Nadu, India. Further school permission was obtained from the Chief Educational Officer of the (CEO) of Thiruvallur district and the Headmistress from Government Girls Higher Secondary School at Keelmanambedu and Government Girls Higher Secondary School Poonamallee. In this study, there were no potential risks that might cause any harm to the adolescent girls. All the information about the adolescent girls was kept private and confidential.

#### 2.3.1 Informed consent from the adolescent girls

All the adolescent girls were given a verbal explanation about the research and were asked to sign the prepared informed consent form made in the local Tamil language. The consent form had information about the study participant, goals of the study, data collection procedure, selection of risk adolescent girls, potential risks, potential benefits, confidentiality of the study participant, voluntary involvement in the study, right to withdraw information, and the contact information, which was explained clearly. This consent form was developed by the researcher and was corrected by the research supervisor. Initially, the consent has been given to three girls and was validated for the ease of understanding the contents.

#### 2.3.2 Informed consent from the parents

A written informed letter was obtained from the parents of the school girls during which they were clarified that the information provided would strictly be anonymous and kept confidential. For any clarification about the study, the principal investigator's contact details were provided.

#### 2.3.3 The right to fair treatment

School girls were selected based on the research requirements and not based on vulnerability, and they had an option to easily approach the research investigator at any point in the study period to clarify their doubts. Written permission was obtained from the girls who were voluntarily ready to provide the pictures. In addition, written permission was also obtained from their parents.

### 2.4 Sample size determination

The sample size was calculated using a pilot study report. Sample size determination was calculated using power analysis.

To reduce the risk from 25% to 10% with *α* = 5%, *β* = 20%


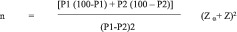


P1 = 25%; P2 = 10%; *α* = 1.96 (allowable error 5%); *β* = 0.84 (power of the study 80%); *d* = 15%.


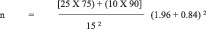


=97 per group = (1875 + 900) (7.84) /225.

**FIGURE 1:**
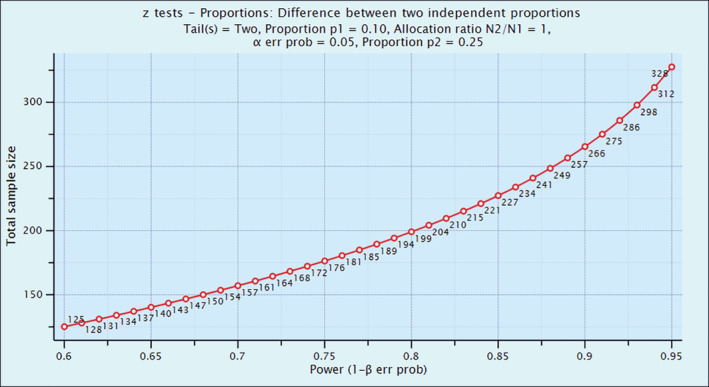
Power analysis of the sample population

The sample size was estimated with the reduction of 15% of risk from baseline (25% of risk), 80% power of the study, 5% allowable error, and 10% of dropout rate. The required sample size 97 + 10 = 107 per group was estimated. Power analysis is illustrated in Figure 1.

### 2.5 Sampling techniques

Nonprobability purposive sampling was used to select the samples. There are a total of 10 Government Girls Higher Secondary Schools functioning in Thiruvallur district. Among these 10 schools, two of them were selected using simple random techniques of lottery method. Out of these two, the first one was chosen for the experimental group (with intervention) and the second one was chosen for the control group. Then, the risk assessment questionnaire was distributed to all the students who fulfilled the inclusion and exclusion criteria of sampling. From these students, “high risk” and “moderate risk” girls were segregated by applying the risk assessment scores developed by Shoba et al.^26^

### 2.6 Sample selection

Both the schools had a total of 1262 students who were studying in the 10th to 12th grade (506 in the experimental group and 756 in the control group). Of these, 442 students fell under the selection criteria in the experimental group (Keelmanambedu School) and 573 in the control group (Poonamallee School). Among these students, sample stratification (segregating only the moderate and high‐risk category group) was done, and the girls with the risk were 105 in the experimental group and 120 in the control group. The remaining adolescent girls fell under the “no risk” and “low‐risk” categories. Of these 102 girls, three girls in the experimental group dropped out from the study as they failed to complete all the follow‐ups and missed the interventions (yoga and exercise).

**FIGURE 2:**
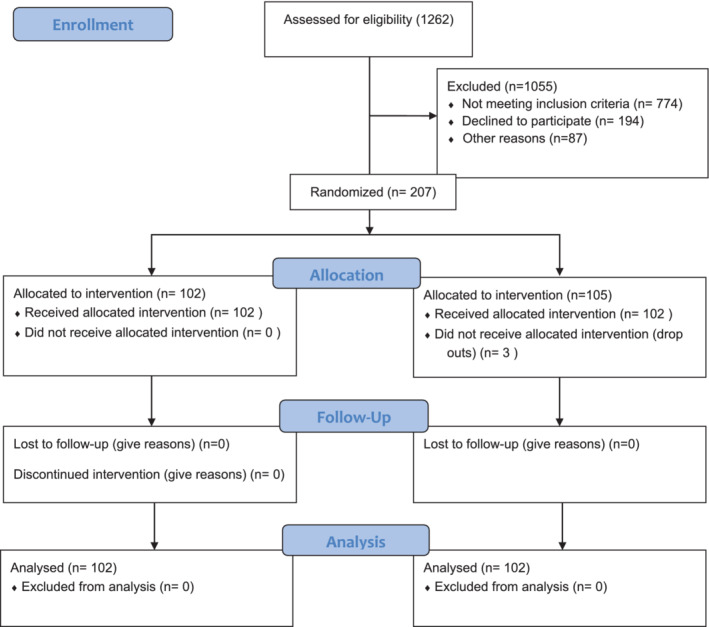
CONSORT flow diagram

From the control group, 102 samples were selected randomly from 120 samples for equalizing the sample size in both the groups. Thus, the total number of subjects who were included for data analysis was n = 102 in the experimental group and n = 102 in the control group. The sample selection is depicted in Figure 2.

### 2.7 Inclusion criteria

Inclusion criteria were adolescent girls studying in 10th to 12th grades who attained menarche before six months, present at the time of data collection, can read, speak, and understand Tamil or English language, and identified with “moderate” and “high risk” for PCOS by using the risk assessment questionnaire.

### 2.8 Exclusion criteria

Exclusion criteria were adolescent girls who were not willing to fill the questionnaire, not willing to participate in lifestyle modification interventions, sick at the time of data collection, practicing yoga and exercise regularly, diagnosed with PCOS, and undergoing treatment were excluded.

### 2.9 Risk assessment questionnaire

The contents of the questionnaire were obtained by the researcher based on the tool originally designed by Shoba et al.^26^ This questionnaire had a total of 16 questions. In that, there were 13 “yes” or “no” type questions and three “rating scale” type of statements. In addition, it also had four demographics parameters. Prior to using the questionnaire, written permission was obtained from the author.

#### 2.9.1 Modification of the questionnaire

After getting permission from the author, the researcher modified 13 “yes” or “no type” questions into rating scale (Q7, Q8, Q9, Q10, Q11, Q12, Q13, Q14, Q15, Q16, Q17, and Q 20). The researcher included three rating scale questions in addition to the ones made by the original author (Q6, Q18, and Q19). In addition, the researcher added four statements (Q16, Q18, Q19, and Q20) based on the inputs obtained from the panel of experts that consisted of Nursing professional, Gynecologist, Psychologist, and Biostatistician. The final risk assessment questionnaire had a total of 20 statements.

Apart from the 20 statements related to PCOS, the authors added 16 demography related questions, such as age, class of study, residence, type of family, religion, number of siblings in the family, order of birth among the siblings, nature of food eating, mode of transportation, age at menarche, nature of food eating, father's and mother's education, occupation of father and mother, monthly income, and sources of information on PCOS.

#### 2.9.2 Risk assessment scoring

The interpretation of the score in percentage is given in Table 1.

**TABLE 1:** Risk assessment score interpretation

Level of risk	Score	Percentage population falling under each category (%)
No risk	0‐10	0‐17
Low risk	11‐20	17.1‐33.3
Moderate risk	21‐40	33.4‐66.6
High risk	41‐60	66.7‐100

While performing risk assessment, the Body Mass Index (BMI) was calculated using the following formula:


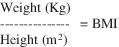


### 2.10 Validation of the study questionnaire

#### 2.10.1 Content validity

The content validity was obtained from the experts from the field of Nursing, Gynecologist, Medical, Sociologist, Psychologist, and Statistician. Face validity was obtained from five subjects. A checklist has been made and experts were asked to fill their responses. The filled responses were analyzed to finalize the contents of the risk assessment and demographic data. The entire questionnaire was also validated based on adolescent girls' responses on their “ability to read” and “ability to understand”.

#### 2.10.2 Reliability

It is a condition in which the same results will be achieved whenever the same technique is repeated within the study.^27^ The reliability was calculated by using Cronbach method. The Cronbach “α” value of risk assessment is 0.86.

#### 2.10.3 Final questionnaire

The final validated questionnaire had 20 questions related to risk assessment on PCOS. The responses used were divided into four categories, where three represented the highest score of “usually”, 2 represented “sometimes”, 1 represented “rarely”, and the lowest score denotes “none” (Appendix S1).

## 3 DATA COLLECTION PROCEDURE

### 3.1 Selection of moderate and high‐risk adolescent girls

#### 3.1.1 Experimental group

The school selected for the experimental group was Government Girls Higher Secondary School, Keelmanambedu. In this school, 506 adolescent girls were studying. Out of the 506 girls, 442 were selected for the study based on the selection criteria. The risk assessment questionnaire was given to each class section‐wise. After the risk assessment, the subject that was filtered for the group which came under “no risk, low risk” category was not included in this study. Of the 442 girls, 90 adolescents were grouped under the category “moderate” and 15 girls were grouped under the “high risk” category. Of the 105 (90 + 15 = 105) adolescent girls, three dropped out during the intervention. The remaining 102 girls completed the entire study, and the data of these 102 girls are included in the result analysis.

#### 3.1.2 Control group

The school that was selected for the control group was Girls Higher Secondary School, Poonamallee, Tamil Nadu, which consists of 756 adolescent girls studying at present. Out of the 756 adolescent girls, 573 were selected for the study based on the selection criteria. The risk assessment questionnaire was given to each class section‐wise. After the risk assessment, subjects were filtered and the group that came under “no risk,” “low risk” category was not included in the study. Of the 573 girls, 103 adolescent girls were grouped under the category “moderate” and 17 girls were grouped under “high risk” category. Of the 120 (103 + 17 = 120) girls, 102 girls were selected randomly in order to equalize the experimental group. One hundred and two adolescent girls completed the entire study and the data of these 102 girls are included in the result analysis (Table 2).

**TABLE 2:** Selection process of moderate and high‐risk subjects

Sample selection	Experimental group	Control group
Total girls from 10th to 12th grade	506	756
Based on selection criteria	442	557
No risk, low risk (excluded)	224 + 113 = 337	265 + 172 = 437
Moderate and high risk (included)	90 + 15 = 105	103 + 17 = 120 (randomization 102)
Drop out	3	No drop out
Final sample for analysis	102	102

## 4 ADMINISTRATION OF QUESTIONNAIRES

The risk assessment and the demographic questionnaire were given to the adolescent girls as a pretest on risk assessment in both the experimental and the control groups. Of the selected girls, the ones categorized under “moderate risk” and “high risk” of PCOS were included, and “low risk” and “no risk” girls were excluded.

The time duration for filling the questionnaires ranged from 30 minutes to 1 hour. After data collection, the researcher held feedback meetings to identify and resolve any problems encountered while filling the questionnaire. The researcher used such meetings as an opportunity for controlling the quality of information by checking the questionnaires for completeness and missing data.

The questionnaire was again administered to both the control and intervention groups' girls after completion of Phase I intervention (yoga) and again after completion of Phase II intervention (Exercise) as described below.

## 5 INTERVENTION

The intervention had two phases:

### 5.1 Phase I intervention (yoga sessions for 2 months)

#### 5.1.1 Conceptualization and development of the yoga training module

The concept of yoga intervention was taken from the traditional yoga scriptures of Patanjali yoga sutras. All interventions were related to the holistic health approach. The Yogasanas consisted of Pranayama, Meditations, Bhadrasana (Butterfly pose), and Chakki Chalanasana (moving the grinding wheel). These specific interventions of yoga were developed by a team of experts, which included Gynecologist, yoga therapist, and nursing professionals.

In order to train the girls for the yoga intervention, the researcher also underwent the training program and got certified from “Legends Yoga training Academy,” Porur, Chennai.

For a better understanding of yoga training as an intervention for the adolescent girls, it has been divided into various stages as given below:

##### *Stage I—Preliminary phase to create awareness among stakeholders*

In order to initiate yoga training and to get the cooperation and to improve awareness among the girls, a yoga training program was organized by the researcher in cooperation from Sri Patanjali Maharishi Yoga Training Centre, Mangadu, Chennai. The program started at 1.30 pm and ended at 4 pm. All the selected girls and even school teachers participated in this program. All the girls sat on the school ground and the school provided the audio facility for the event.

In this session, the trainer gave a 1‐hour lecture that included an introduction to the importance of yoga and the benefits of yoga in PCOS. Following this, for the next 1.5 hours, the entire adolescent girls were trained on the asanas. Then, the adolescent girls were requested to come at 8.30 am for practicing yoga for two months.

##### *Stage II—Performance of yoga by the adolescent girls*

On the first‐day, adolescent girls were made to gather on the ground under a tree on the side of the school and practiced only meditation and pranayama, and on the second day they practiced butterfly pose and chakki chalasana pose. From the third day onward, all the four asanas were practiced by the girls.

##### *Stage III—Continuation phase*

From the third day onward, a 1‐minute introduction was given daily by the investigator about yoga, and the girls were motivated to continue yoga. The girls were made to do meditation for 5 minutes then relaxation for 2 minutes, pranayama for 5 minutes then 2 minutes for relaxation, 5 minutes for butterfly asana then 2 minutes for relaxation, chakki chalasana asana for 5 minutes then 2 minutes relaxation finally at the end of the interventions.

##### *Stage IV—Sustenance phase*

After two months of yoga intervention, adolescent girls were made to do brisk walking exercise for 30 minutes daily. They were advised to come to school at 8.30 am regularly for 2 months. Log book was maintained by the researcher. Adolescent girls were advised to do yoga even during the holidays.

*Note*: As an ethical point of view, yoga was taught to the control group at the end of the study.

### 5.2 Phase II intervention (exercise sessions for 2 months)

#### 5.2.1 Stage 1—Preliminary phase

After reviewing various journals and books, as well as expert opinion, the researcher incorporated to do brisk walking exercises for the girls after the yoga intervention in the experimental group.

#### 5.2.2 Stage II—Performance phase

Girls were requested to come regularly for practicing walking exercise for 2 months in the school ground at 8.30 am daily. The researcher also maintained a log with the help of the school teachers and class representative of each grade. The absentees were requested to do walking exercise on Sunday to compensate the absence, since walking exercise was planned for weekly six days. All of them were also requested to continue the intervention on Government holidays.

*Note*: As an ethical point of view, brisk walking exercise was taught for the adolescent girls in the control group at the end of the study.

## 6 DATA PROCESSING AND ANALYSIS

The collected data were checked for consistency and completeness, and it was analyzed using Statistical Package for Social Sciences (SPSS, version 16) and STATA (version 12) software and Epi info (version 3.5.1).

### 6.1 Statistical analysis

#### 6.1.1 Descriptive statistics

Frequencies, means, and SDs were used to analyze the demographic characteristics. Quantitative risk score was given in mean and SD. Quantitative risk, with respect to different grades of scoring, was presented using frequency and percentages. The similarity of demographic variables, distribution between the experiment and the control was tested using Chi‐square test.

#### 6.1.2 Inferential statistics

The pretest and posttest mean risk score results were compared using the paired *t* test to evaluate the impact of lifestyle modifications on the risk for PCOS among adolescent girls. Pretest, posttest‐1, and posttest‐2 statistical differences are found.

### 6.2 Pilot study

Pilot study was conducted in Government Girls Higher Secondary School, Kamaraj Nagar, Avadi, Thiruvallur district.^28^ The following modifications were carried out based on the pilot study's findings:

1. A question (Q9. How do you normally come to school?) was added in the demographic variables.
2. Initially, six yogasanas were planned and executed for the pilot study. However, considering the time factor and the suggestions by the experts, four yogasanas, namely “pranayama”, “meditations”, “bhadrasana”, and “chakki chalanasana” were executed in the main study, and two yogasanas namely “bharadvajasana” and “sun salutation” were excluded from the main study.

## 7 RESULTS

### 7.1 Demographic profile of the study respondents

Altogether 204 (experimental, n = 102; control, n = 102) samples were enrolled in the research as per the inclusion and exclusion criteria of the study. Table 3 shows the demographic features of the study subjects.

**TABLE 3:** Demographic profile of the study respondents in both the groups (n = 204)

Demographic variables		Group				Chi‐square test
		Experiment (n = 102)		Control (n = 102)		
		n	%	n	%	
Age (in years)	15 16 17 and above	28 45 29	27.5 44.1 28.4	21 53 28	20.6 52.0 27.4	*χ*2 = 1.67 *P* = .43 DF = 2 NS
Class	10th grade 11th grade 12th grade	34 39 29	33.3 38.3 28.4	29 41 32	28.4 40.2 31.4	*χ*2 = 0.59 *P* = .74 DF = 2 NS
Residence	Rural Semi‐urban Urban	36 37 29	35.3 36.3 28.4	28 42 32	27.4 41.2 31.4	*χ*2 = 1.46 *P* = .48 DF = 2 NS
Type of family	Nuclear Joint Extended	71 26 5	69.6 25.5 4.9	78 20 4	76.5 19.6 3.9	*χ*2 = 1.22 *P* = .54 DF = 2 NS
Religion	Hindu Christian Muslim	87 9 6	85.3 8.8 5.9	80 14 8	78.4 13.8 7.8	*χ*2 = 1.66 *P* = .43 DF = 2 NS
Sibling	None One Two > Two	3 50 35 14	2.9 49.0 34.3 13.8	3 44 42 13	2.9 43.1 41.3 12.7	*χ*2 = 1.05 *P* = .79 DF = 3 NS
Order of child	Eldest Second Third Fourth or above	37 41 21 3	36.3 40.2 20.6 2.9	36 36 28 2	35.3 35.3 27.4 2.0	χ2 = 1.59 *P* = .67 DF = 3 NS
Age at Menarche (in years)	11 12 13 14 15 and above	9 33 36 19 5	8.8 32.4 35.3 18.6 4.9	10 35 23 26 8	9.8 34.3 22.5 25.6 7.8	*χ*2 = 4.7 *P* = .31 DF = 4 NS
Type of food	Vegetarian	24	23.5	21	20.6	*χ*2 = .25 *P* = .61 DF = 1 NS
	Nonvegetarian	78	76.5	81	79.4	
Mode of transportation	Walking	24	23.5	25	24.5	*χ*2 = 0.25 *P* = .61 DF = 3 NS
	Bus	71	69.7	67	65.7	
	Cycling	4	3.9	5	4.9	
	Others	3	2.9	5	4.9	
Source of information	No information	11	10.8	22	21.6	*χ*2 = 0.74 *P* = .86 DF = 5 NS
	Television	41	40.2	32	31.4	
	News paper	25	24.5	16	15.7	
	Friends	8	7.8	13	12.7	
	Health personnel	15	14.7	17	16.6	
	Books	2	2.0	2	2.0	

*Note*: NS, No significant; DF, degrees of freedom. No significant *P* > .05.

### 7.2 Pretest level of risk assessment

**FIGURE 3:**
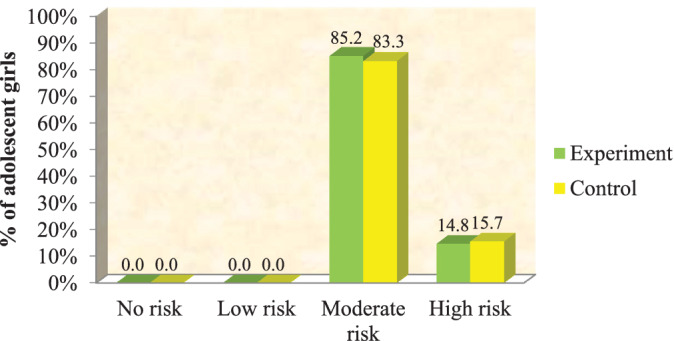
Pretest level of risk assessment in both the groups (n = 204). *Note*:  Chi‐square test = 0.15 DF = 1 *P* = .70 (no significant *P* > .05)

There was no significant difference between the PCOS risk assessment score of participants in the experimental and the control groups (*P* = .70; *χ*2 = 0.15). Figure 3 shows the pretest level of risk on PCOS among the adolescent girls in the experimental and control groups.

### 7.3 Comparison of the experimental group's and the control group's level of risk assessment score for pretest, posttest‐1, and posttest‐2

There was no significant difference between the PCOS risk assessment score of participants in the experimental and control groups (*P* = .70; *χ*2 = 0.15). In both the groups, none of the adolescent girls had either “no risk” or “low risk level.” Table 4 reveals the risk of PCOS among adolescent girls in the experimental and the control groups during pretest, posttest‐1, and posttest‐2.

**TABLE 4:** Comparison of the experimental group's and the control group's level of risk assessment score for pretest, posttest‐1, and posttest‐2 (n = 204)

Group	Risk	Group				Chi‐square test
		Experiment		Control		
		n	%	n	%	
Pretest	Moderate risk	87	85.2	85	83.3	χ2 = 0.15 *P* = .70 DF = 1
	High risk	15	14.8	17	15.7	
	Total	102	100.0	102	100.0	
Posttest‐1	No risk	10	9.8	0	0.0	χ2 = 27.15 *P* = .001*** DF = 3
	Low risk	13	12.7	0	0.0	
	Moderate risk	73	71.6	89	87.3	
	High risk	6	5.9	13	12.7	
	Total	102	100.0	102	100.0	
Posttest‐2	No risk	21	20.6	0	0.0	χ2 = 77.09 *P* = .001*** DF = 2
	Low risk	32	31.4	0	0.0	
	Moderate risk	48	48.0	90	88.2	
	High risk	0	0.0	12	11.8	
	Total	102	100.0	102	100.0	

*Note*: Not significant *P* > .05 *** very high significant at *P* ≤ 0.001.

### 7.4 Comparison of pretest, posttest‐1, and posttest‐2 risk assessment score in both the groups

Table 5 shows that repeated measure ANOVA with Greenhouse‐Geisser correction showed that mean risk reduction score difference (10.63) was statistically significant (*F* = 236.12 *P* < .001) between pretest (33.38 ± 7.28) and posttest (22.75 ± 12.09) in the experimental group's adolescent girls.

**TABLE 5:** Six comparison of pretest, posttest‐1, and posttest‐2 risk assessment score in both the groups (n = 204)

Group	Pretest		Posttest‐1		Posttest‐2		Mean difference	Repeated measures ANOVA *F*‐test
	Mean	SD	Mean	SD	Mean	SD		
Experiment	33.38	7.28	27.20	10.21	22.75	12.09	10.63	*F* = 236.12 *P* = .001***
Control	34.84	6.37	34.52	8.06	34.22	6.93	0.62	*F* = 1.16 *P* = .82

### 7.5 Comparison of the experimental group's and control group's level of BMI

Table 6 shows the BMI of adolescent girls in the experimental group and the control group during pretest, posttest‐1, and posttest‐2. Statistically, there was no significant difference found between the BMI of the experimental group and the control group in pretest.

**TABLE 6:** Comparison of the experimental group's and the control group's level of BMI (n = 204)

Test	Weight status	Group				Chi‐square test
		Experiment		Control		
		n	%	n	%	
Pretest	Underweight	10	9.8	13	12.7	*χ*2 = 0.71 *P* = .87 DF = 3 not Significant
	Normal	68	66.7	66	64.7	
	Overweight	22	21.6	20	19.6	
	Obese	2	1.9	3	3.0	
	Total	102	100.0	102	100.0	
Posttest‐1	Underweight	8	9.8	12	11.8	χ2 = 2.01 *P* = .57 DF = 3 Significant
	Normal	76	71.6	67	65.7	
	Overweight	16	12.7	20	19.6	
	Obese	2	5.9	3	2.9	
	Total	102	100.0	102	100.0	
Posttest‐2	Underweight	6	6.0	12	11.8	χ2 = 7.62 ***P* =** .05* DF = 3 Significant
	Normal	83	81.3	68	66.7	
	Overweight	13	12.7	19	18.6	
	Obese	0	0.0	3	2.9	
	Total	102	100.0	102	100.0	

*Note*: Not significant *P* > .05 * significant at *P* ≤ .05 ** highly significant at *P* ≤ .01 *** very high significant at *P* ≤ .001.

### 7.6 Association between risk assessment score and adolescent girls' demographic variables in experimental group

Table 7 depicts the association between risk assessment score of the experimental group's adolescent girls and their demographic variables. With reference to the above table, only three variables, namely age (*F* = 3.28 at *P* = .05), class (*F* = 3.30 at *P* = .05), and order of birth (*F* = 3.13 at *P* = .03), were significantly associated with their risk assessment score. But there was no statistically significant association observed between the rest of the variables, such as place of residence (*F* = 1.13 at *P* = .05), type of family (*F* = 0.47 at *P* = .62), religion (*F* = 0.71 at *P* = .49), number of siblings (*F* = 0.71 at *P* = .54), age at menarche (*F* = 0.90 at *P* = .44), with their risk assessment score.

**TABLE 7:** Association between risk assessment score and adolescent girls' demographic variables in experimental group (n = 102)

Demographic variables		n	Risk assessment score						
			Pretest		Posttest		Reduction score = pre‐post		One‐way
			Mean	SD	Mean	SD	Mean	SD	ANOVA *F*‐test/*t*‐test
Age (in years)	15	28	34.32	6.80	25.86	11.60	11.46	7.49	*F* = 3.28 *P* = .05* S
	16	45	33.24	7.19	22.96	12.11	13.49	7.47	
	17 and above	29	32.69	7.97	19.79	12.72	16.48	7.42	
Class	10th grade	34	34.38	6.30	26.18	11.36	11.21	7.76	*F* = 3.30 *P* = .05* S
	11th grade	39	32.90	7.70	21.85	12.62	14.51	7.69	
	12th grade	29	32.86	7.86	20.31	12.29	15.83	6.74	
Place of residence	Rural	36	32.06	6.89	20.22	12.60	15.28	7.72	*F* = 1.13 *P* = .33 (NS)
	Semi‐urban	37	35.11	8.37	25.46	11.79	12.68	7.13	
	Urban	29	32.83	5.92	22.79	12.07	13.34	8.09	
Type of family	Nuclear	71	33.39	7.51	22.41	11.92	14.13	7.22	*F* = 0.47 *P* = .62 NS
	Joint	26	32.42	5.56	22.35	12.89	13.42	8.89	
	Extended	5	38.20	11.03	31.80	12.40	10.80	6.94	
Religion	Hindu	87	33.52	7.44	22.95	12.34	13.86	7.60	*F* = 0.71 *P* = .49 NS
	Christian	9	32.00	7.62	19.78	11.82	15.22	7.00	
	Muslim	6	33.50	4.42	26.00	12.52	10.50	9.35	
Number of siblings	None	3	30.00	4.58	21.67	14.19	11.33	9.61	F = 0.71 *P* = .54 NS
	One	50	34.32	6.31	24.90	11.59	12.60	7.48	
	Two	35	33.31	8.19	22.86	12.33	13.49	7.59	
	>Two	14	30.93	8.36	15.79	12.64	19.29	6.08	
Order of birth	Eldest	37	35.11	7.12	22.19	11.96	12.92	7.22	*F* = 3.13 *P* = .03* (S)
	Second	41	36.10	5.08	19.69	11.39	16.41	7.99	
	Third	21	34.86	10.11	21.67	14.02	13.19	7.38	
	Fourth and above	3	33.00	6.24	20.33	15.89	12.67	9.87	
Age at Menarche (in years)	11	9	33.67	6.02	22.89	11.48	13.78	6.14	*F* = .90 *P* = .44 (NS)
	12	33	34.70	8.13	23.85	13.23	14.36	8.20	
	13	36	32.42	7.84	21.64	12.41	13.78	7.22	
	14	19	32.63	5.34	23.16	11.34	12.47	7.37	
	15 and above	5	34.00	6.60	23.80	13.08	15.00	11.94	
Type of food	Vegetarian	24	33.17	6.14	22.04	12.54	14.88	8.74	*t* = 0.80 *P* = .43 NS
	Nonvegetarian	78	33.45	7.63	23.10	12.22	13.45	7.28	
Mode of transportation	Walking	24	33.58	5.17	24.42	11.28	12.54	8.18	*F* = 2.38 *P* = .07 NS
	Bus	71	33.61	7.97	23.32	12.32	13.52	7.35	
	Cycling	4	28.25	1.50	9.00	3.92	22.25	2.63	
	Others	3	33.33	9.24	17.67	17.62	18.67	8.39	
Source of information	No information	11	33.45	6.33	24.45	12.36	12.00	7.13	*F* = 1.09 *P* = .37 NS
	Television	41	34.29	7.77	23.41	12.04	13.90	7.05	
	News paper	25	33.92	7.10	25.16	12.53	12.12	8.43	
	Friends	8	33.00	9.77	19.75	14.95	17.13	8.51	
	Health person	15	30.67	5.78	18.00	11.05	16.27	7.20	
	Books	2	29.50	2.12	22.50	13.44	10.00	11.31	

*Note*: NS, not significant; S, significant not significant *P* > .05 at * significant at *P* ≤ .05.

### 7.7 Association between risk assessment score and demographic variables in the control group

Table 8 shows the association between the risk assessment score and adolescent girls demographic variables in the control group. None of the demographic variables like “age” (*F* = 0.59 at *P* = .55), class (*F* = 1.75 at *P* = .17), place of residence (*F* = 3.53 at *P* = .03), type of family (*F* = 1.62 at *P* = .20), religion (*F* = 0.62 at *P* = .945), number of siblings (*F* = 1.73 at *P* = .16), order of birth (*F* = 1.14 at *P* = .34), age at menarche (*F* = 0.60 at *P* = .66), type of family (*t* = 1.99 at *P* = .08), mode of transportation to school (*F* = 2.24 at *P* = .08), and source of information (*F* = 1.12 at *P* = .38) had any significant association with the attitude score.

**TABLE 8:** Association between risk assessment score and demographic variables in the control group (n = 102)

Demographic variables		n	Risk assessment score						
			Pretest		Posttest		Gain score = post‐pre		One‐way
			Mean	SD	Mean	SD	Mean	SD	ANOVA *F*‐test/*t*‐test
Age	15 years	21	34.57	5.80	31.14	8.34	3.43	3.67	*F* = 0.59 *P* = .55 NS
	16 years	35	34.60	7.05	32.89	10.25	1.72	5.91	
	17 years and more	24	35.50	5.51	33.14	10.36	2.36	7.71	
Class	10th grade	26	33.69	5.70	30.55	8.67	3.14	4.17	*F* = 1.75 *P* = .17 NS
	11th grade	30	35.37	7.69	34.49	10.84	0.88	5.91	
	12th grade	24	35.22	4.98	32.03	9.38	3.19	7.46	
Place of residence	Rural	28	35.39	6.58	34.86	9.91	0.54	6.41	*F* = 3.53 *P* = .03 NS
	Semi urban	29	34.10	6.70	30.07	9.86	4.02	5.99	
	Urban	23	35.34	5.81	33.94	9.34	1.41	5.44	
Type of family	Nuclear	53	34.71	6.44	32.86	10.19	1.85	6.48	*F* = 1.62 *P* = .20 NS
	Joint	23	35.20	6.70	32.40	8.88	2.80	3.91	
	Extended	4	35.75	3.59	28.50	8.81	7.25	5.50	
Religion	Hindu	69	35.23	6.65	32.88	9.87	2.35	6.18	*F* = 0.06 *P* = .945 NS
	Christian	6	33.07	5.58	31.21	11.03	1.86	6.26	
	Muslim	5	34.13	4.36	32.25	8.46	1.88	5.49	
Number of siblings	None	2	35.00	10.15	37.33	13.01	−2.33	3.21	*F* = 1.73 *P* = .16 NS
	One	41	35.11	6.21	32.14	9.53	2.98	5.84	
	Two	26	34.48	6.03	31.83	10.35	2.64	6.64	
	>Two	11	35.08	7.80	35.54	8.92	−.46	4.59	
Order of birth	Eldest	32	33.75	4.69	30.81	9.30	2.94	6.81	*F* = 1.14 *P* = .34 NS
	Second	31	35.67	5.81	32.89	9.53	2.78	6.06	
	Third	14	34.96	8.51	33.93	10.76	1.04	5.10	
	Fourth or above	3	38.00	9.90	41.00	12.73	−3.00	2.83	
Age at Menarche (in years)	11	9	35.10	4.31	30.60	7.68	4.50	5.19	*F* = 0.60 *P* = .66 NS
	12	26	35.83	6.09	34.37	10.14	1.46	6.51	
	13	26	32.57	5.95	29.91	6.47	2.65	4.15	
	14	14	36.04	7.39	34.27	12.12	1.77	7.66	
	15 and above	5	32.88	6.53	29.63	10.14	3.25	4.10	
Type of food	Vegetarian	20	33.95	5.04	29.38	6.93	4.57	5.23	*t* = 1.99 *P* = .05 NS
	Nonvegetarian	60	35.07	6.68	33.43	10.35	1.64	6.17	
Mode of transportation	Walking	18	36.56	7.06	36.64	10.23	−.08	6.01	*F* = 2.24 *P* = .08 NS
	Bus	55	34.51	6.27	31.60	9.57	2.91	6.02	
	Cycling	4	33.20	4.15	32.00	10.68	1.20	7.29	
	Others.	3	32.40	5.18	26.40	5.55	6.00	2.65	
Source of information	No information	11	34.05	5.52	32.32	10.81	1.73	6.80	*F* = 1.12 *P* = .38 NS
	Television	30	35.31	7.44	32.47	9.95	2.84	5.18	
	News paper	18	35.00	7.04	31.75	7.66	3.25	4.65	
	Friends	7	35.92	6.18	36.77	8.41	−.85	4.96	
	Health personnel	12	34.76	5.04	32.12	11.32	2.65	8.34	
	Books	2	28.50	3.54	21.50	3.54	7.00	0.00	

*Note*: NS = not significant not significant *P* > .05.

### 7.8 Identification of influencing factors for risk assessment score using multivariate logistic regression

Table 9 shows the multivariate logistic regression analysis, which identifies 15‐year‐old girls, 12th grade girls, more educated mothers, and family income of more than Rs. 5000 for adolescent girls have reduced more risk score than others after intervention. Adjusted odds ratio was given with 95% confidence interval.

**TABLE 9:** Identification of influencing factors for risk assessment score using multivariate logistic regression

Demographic variables	Univariate analysis		Multivariate analysis	
	*P* value	UnadjustedOR(95%CI)	*P* value	AdjustedOR(95%CI)
Age (≥15 years vs < 15 years)	.02*	2.0 (1.0‐7.7)	.05*	1.8(1.1‐4.1)
Class (≥10th grade vs < 10th grade)	.05*	3.0 (1.1‐7.8)	.05*	1.7(0.7‐2.3)
Birth order (≤Second vs > Second)	.02*	3.1(1.0‐9.5)	.53	1.1(0.6‐2.1)
Mother's education (≥ primary vs < primary)	.01**	2.8 (1.2‐6.9)	.03*	1.9(1.1‐4.8)
Monthly income (≥Rs.5000 vs ≤ Rs.5000)	.02*	2.7 (1.1‐6.8)	.02*	1.6(1.0‐5.2)

Table 10 shows the question‐wise percentage of risk assessment score on PCOS among the experimental group and the control group of adolescent girls during pretest to posttest‐2. Upon a maximum score of 60, the mean risk assessment score was reduced by 17.7% in the experimental group after intervention from a pretest score of 55.6% to a posttest‐2 score of 37.9%. However, the mean risk assessment score was reduced by 1.1% only in the control group from a pretest score of 58.1% to a posttest‐2 score of 57%.

**TABLE 10:** Each question‐wise percentage of risk assessment score in both the groups (n = 204)

Questions	Max score	Experiment			Control		
		Pretest	Posttest‐2	% gain score	Pretest	Posttest‐2	% gain score
Do you have irregular periods?	3	74.3	57.7	16.6	76.0	74.7	1.3
Do you miss your period in your regular cycle?	3	50.3	38.0	12.3	53.3	51.7	1.6
Do you get your menstrual cycle after more than 35 days?	3	40.7	33.7	7.0	43.7	42.7	1.0
Do you have your menstrual flow for more than five days?	3	54.7	42.7	12.0	56.0	55.7	0.3
Are you changing more than 4 pads per day?	3	62.3	49.7	12.6	64.3	63.3	1.0
Do you have the habit of eating junk food?	3	75.0	48.7	26.3	77.7	76.0	1.7
Do you experience nausea/vomiting during menstruation?	3	34.3	28.0	6.3	38.3	37.3	1.0
Do you experience abdominal pain during menstruation?	3	72.0	41.7	30.3	75.0	72.3	2.7
Do you experience pain/tenderness in breast during menstruation?	3	41.0	27.3	13.7	42.7	43.0	−0.3
Do you have heavy menstrual flow with clots?	3	59.7	39.7	20.0	62.3	60.3	2.0
Do you have pimples on your face?	3	65.3	37.7	27.6	67.7	66.0	1.7
Do you have hair fall?	3	72.3	50.3	22.0	74.0	73.3	0.7
Do you have problem with hair growth over chest, face or abdomen?	3	16.0	15.0	1.0	18.7	18.7	0.0
Do you have any darkening and thickening of skin folds around the neck and axillae?	3	45.3	38.0	7.3	46.7	46.7	0.0
Do you have weight gain?	3	57.0	39.3	17.7	58.7	58.7	0.0
Do you have difficulty in losing weight?	3	52.7	36.0	16.7	55.3	54.7	0.6
Do you have frequent thirst and urination?	3	59.0	36.3	22.7	62.0	60.0	2.0
Do you feel extremely hungry, irritable and sleepy?	3	58.7	34.3	24.4	61.0	61.0	0.0
Do you have symptoms of giddiness and fatigue?	3	59.7	27.7	32.0	61.7	61.0	0.7
Do you have stressful and depressive mind?	3	62.3	36.7	25.6	64.7	63.0	1.7
Total	60	55.6	37.9	17.7	58.1	57.0	1.1

## 8 DISCUSSION

PCOS affects women's health in multiple ways, ranging from sense of well‐being to impaired reproductive health. Risk assessment for PCOS can largely help early identification and providing better remedies. In this research, a modified version of a standard PCOS risk assessment questionnaire was used to assess the PCOS risk among adolescent school going girls. This assessment mainly focused on their menstruation, eating habits, excessive hair growth, junk foods, and the psychological factors, such as depression and stress. The impact of intervention on the PCOS risk was studied, and the findings showed a reduction in the risks, such as the BMI. In one study, authors found menstrual cycle disorder, bad mood, family history of diabetes, and family history of infertility menstrual irregularity of mother, and lack of physical exercise as risk factors for PCOS.^29^ Obesity, central obesity, and insulin resistance are strongly implicated in its etiology, and reduction of these risk factors should be the central focus of the treatment. Short‐term weight loss has been consistently successful in reducing insulin resistance and restoring ovulation and fertility.^30^

In the present research, the mean risk assessment score reduced after yoga and continued to reduce after the exercise sessions. It was important to note that the percentage of adolescent girls with high risk of PCOS decreased to 5.9% in posttest 1 and “none” in posttest 2 in experimental groups. One research showed that yoga helped in controlling endocrine function and decreasing the symptoms of PCOS.^31^ Other reported studies showed significant reduction in triglycerides levels, decrease in luteinizing/follicle stimulating hormone ratio, and the sex hormone binding globulin levels, and improvement following yoga.^32^, ^33^ However, in the present research, no laboratory investigators were performed to assess the impact of intervention, and the assessment was based on a risk assessment tool. Few similar studies reported that lifestyle modification helps, to hormonal profile helps, to restore the ovulation for the initial management of the reproductive disorder^34^, ^35^, ^36^, ^37^ had also showed that exercise plays an important role in the risk reduction of PCOS.

It was noticed that during the pretest in the experimental group, 85.2% of them had moderate risk and 14.8% had high risk level of PCOS. The percentage of adolescent girls with high risk of PCOS decreased to 5.9% in posttest‐1 and none in posttest‐2 in the experimental group. Yoga is a holistic approach toward better health. In PCOS, it is agreed that yoga can be beneficial.^38^ In one research, authors found yoga very effective than even physical exercises in improving glucose, lipid levels, and minimized insulin resistance in adolescent PCOS girls.^8^

It is well known that PCOS is linked with menstrual abnormalities.^39^, ^40^, ^41^ In the present research, 50% of girls in the experimental group responded that they have irregular periods “usually” and 48% responded that they “sometimes” miss their periods in their regular cycle. Half of the participants (50%) responded that they “sometimes” get their menstrual cycle after more than 35 days and 40% responded that they “sometimes” get menstrual flow for more than 5 days. Thirty‐six percentage and 34.3% responded that they change more than four pads per day “sometimes” and “usually,” respectively. Similar to the test group, even in the control group, a greater number of the girls, 52%, responded that they have irregular periods “usually” and 48% responded that they “sometimes” miss their periods in their regular cycle. Fifty‐two percentage of the participants responded that they “sometimes” get their menstrual cycle after more than 35 days and 42.2% responded that they “sometimes” get menstrual flow for more than five day. Thirty‐nine percentage and 34.3% responded that they change more than 4 pads per day “sometimes” and “usually,” respectively. In a study from India, 6.3% of the girls had oligomenorrhea with PCOS, 0.22% had clinical hyperandrogenism, and 2.39% had oligomenorrhea in the presence of clinical hyperandrogenism.^18^ A similar study found that a greater number of girls had irregular menstrual periods.^42^ This indicates that girls with PCOS risk have irregular menstrual periods. If untreated, this condition can affect the reproductive health and can pose a major problem in their family life. So, it is commonly understood that irregular menstrual period is one of the signs and symptoms of PCOS. Thus, the responsibility lies in the hands of health professionals, school teachers, and parents to find out this problem and help them to treat or prevent PCOS. Upon intervention, there was a slight improvement in the menstrual conditions of the students in the experimental group. After the intervention, they knew the importance of regular menstrual period, and they would consider meeting the doctor for better preventive care.

Eating junk foods can be harmful^43^ and is known to have a link with PCOS.^44^ The present research findings showed a higher number of girls having the habit of eating junk foods. Similarly, higher mean scores of 2.25 and 2.33 were seen for the habit of eating junk food among the experimental group and the control group participants, respectively. Eating junk foods can cause disturbance in carbohydrate metabolism and thus can cause abnormal blood glucose, which is linked with PCOS. A study conducted in India reported a greater number of adolescent girls with PCOS were having the habit of eating junk foods.^26^ The schoolgirls in the experimental group received education on the harmful effects of junk food. The students were encouraged to avoid eating junk foods. Many of the students had the habit of eating junk foods sold in front of the school. They agreed with the researcher that they will try to stop eating as much as possible as it was linked to their reproductive health.

Obesity is a well‐known risk factor for PCOS.^45^, ^46^, ^47^ It is well understood that obese girls carry more risk for developing PCOS in future and hence it is important to help them in devising strategies to overcome obesity and associated problems. In the present research, among experiment group, 21.6% (n = 22) were overweight and 1.9% (n = 2) were obese. In the control group, 19.6% (n = 20) of them were overweight and 3.0% (n = 3) were obese. In various studies, significantly higher number of women had a high BMI, suggesting the relationship between obesity and PCOS.^48^, ^49^, ^50^ Upon intervention, in the present research, students agreed to perform exercise and yoga daily and make it as a day to day habit. They may also spread the importance of performing exercise and yoga to their peer groups. There was also a slight improvement in the BMI of intervention group girls. However, this finding is a short‐term observation and it would be interesting to monitor these girls for a long time and see the impact of the awareness program, exercise, and yoga on their BMI.

PCOS is strongly linked to psychological symptoms^51^ and these symptoms can further influence the diagnosis and treatment of PCOS.^52^ In the present research, at baseline, majority of the girls were “usually” depressive, feeling “irritable and sleepy” giddiness fatigue sometimes in the experimental group and the control group. This could be due to their examinations and other school related stress. However, studies showed that anxiety level and the depression among women with PCOS is more common.^31^, ^52^ If left unattended it can worsen the quality of life, poor academic performance, and can also later lead to chronic psychiatric problems. The schoolgirls in the experimental group received counseling and education to tackle the psychological stress factors. After the intervention, as mentioned in Table 10, there were improvements in the questions related to psychological factors. Most of the girls showed more energy and were relieved from depression. It is also imperative that exercise and yoga can certainly improve the psychological aspects of any individual, as it involves both body and mind.

## 9 LIMITATIONS

This study was limited only to “high” risk and “moderate risk” adolescent girls. “Low risk” and “no risk” adolescents' girls were not included in the study and hence can be a limitation. Furthermore, this research was restricted only to adolescent girls who are studying in 10th to 12th grades in two government Girls Higher Secondary Schools and hence may not be representative for the entire school girls. Private schools were exempted and thus possess inclusion bias. This study was limited only to 4 months of lifestyle modifications and only 30 minutes a day. Having more time could have provided better results. The researcher could not supervise the adolescent girls performing yoga and exercise during government holidays. Self‐reported information relies primarily on respondents providing the right information. Misreporting by respondents cannot be ruled out.

## 10 CONCLUSIONS

This research finding concluded yoga and exercise customized as per school girls schedule involving school teachers can have a positive impact on PCOS risk reduction. As reported in the research, upon interventions, a significant risk reduction occurred among the experimental group, suggesting the usefulness of interventions. This study thus provides a foundation for incorporating lifestyle interventions among adolescent school going girls with PCOS risk. Lifestyle modifications among school going adolescent girls may be a successful strategy in PCOS risk minimization among the Indian population.

## ETHICS STATEMENT

The ethical approval was obtained from the Billroth Hospital Ethical Review Board, Chennai, India.

## AUTHOR CONTRIBUTIONS

Conceptualization: Valarmathi Selvaraj, Jain Vanitha, Fabiola M. Dhanaraj, Prema Sekar, Anitha Rajendra Babu

Data Curation: Valarmathi Selvaraj

Formal Analysis: Valarmathi Selvaraj, Jain Vanitha

Investigation: Valarmathi Selvaraj, Fabiola M. Dhanaraj

Methodology: Valarmathi Selvaraj, Jain Vanitha, Fabiola M. Dhanaraj, Prema Sekar, Anitha Rajendra Babu

Resources: Valarmathi Selvaraj, Fabiola M. Dhanaraj

Supervision: Valarmathi Selvaraj, Jain Vanitha, Fabiola M. Dhanaraj, Prema Sekar, Anitha Rajendra Babu

Validation: Jain Vanitha, Prema Sekar, Anitha Rajendra Babu

Writing – Review & Editing: Valarmathi Selvaraj, Fabiola M. Dhanaraj, Prema Sekar

 All authors have read and approved the final version of the manuscript.

 Valarmathi Selvaraj had full access to all of the data in this study and takes complete responsibility for the integrity of the data and the accuracy of the data analysis.

## FUNDING

This research was self‐financed by the Principal investigator (VS) as a part of her PhD research with her personal money.

## CONFLICT OF INTEREST

The authors declare there is no conflict of interest.

## CONSENT FOR PUBLICATION

The consent is taken from both the study subjects and their parents.

### TRANSPARENCY STATEMENT

Valarmathi Selvaraj affirms that this manuscript is an honest, accurate, and transparent account of the study being reported; that no important aspects of the study have been omitted; and that any discrepancies from the study as planned (and, if relevant, registered) have been explained.

## Supporting information


**Appendix S1**. Supporting Information.

## Data Availability

The data of this research can be obtained from the corresponding author with an Email request.
