# Supplementary material for: Impact of yoga and exercises on polycystic ovarian syndrome risk among adolescent schoolgirls in South India
Source: Health Sci Rep. 2020 Dec 4;3(4):e212. doi: 10.1002/hsr2.212 (PMC7717472; doi:10.1002/hsr2.212)
Supplement: Supplementary file 1 — Appendix S1. Supporting Information. [file HSR2-3-e212-s001.docx]

**Appendix 1**

**Section A**

**Demographic data (English)**

**Class and division Date: School name:**

**____________________________________________________________________**

**Instructions**:

Dear student, kindly go through the questionnaire and put a tick mark ($\surd$ ) on the correct responses.

**1. Your age in years**

a. 15

b.16

c. 17and more specify_______________

**2. Class of study**

a. 10^th^ grade

b. 11^th^ grade

c. 12^th^ grade

**3. Residence**

a. Rural

b. Sub urban

c. Urban

**4. Type of family**

a. Nuclear

b. Joint

c. Extended

**5. Religion**

a. Hindu

b. Christian

c. Muslims

d. Others (please specify) _______________

**6. Number of siblings in your family**

a. None

b. 1

c. 2

d. 3 and more (please specify the number) __________

**7. Order of birth among your siblings**

a. Eldest

b. Second

c. Third

d. Fourth or above (please specify the order) _____________

**8. What nature of food do you eat?**

1. Vegetarian
2. Non-vegetarian

**9. How do you normally come to school?**

a. Walking

b. Bus

c. Cycling

d. Others (please specify) __________________

**10. Age of menarche (in years)**

Mention the age here (in years) ____________

**11. Fathers education**

a. No formal education

b. Primary school

c. Middle school

d. High school

e. Higher secondary school

f. Diploma

g. Graduate

h. Post graduate

**12. Mothers education**

a. No formal education

b. Primary school

c. Middle school

d. High school

e. Higher secondary school

f. Diploma

g. Graduate

h. Post graduate

**13. Occupation of the father**

a. Un employed

b. Daily wages

c. Self employed

d. Semi skilled worker

e. Skilled worker

f. Others (please specify) _______________

**14. Occupation of the mother**

a. Un employed

b. Daily wages

c. Self employed

d. Semi-skilled worker

e. Skilled worker

f. Others (please specify) _______________

**15. Monthly income of the family (in rupees)**

a. Less than3000

b. 3001-5000

c. 5001-7000

d. More than7000

**16. Sources of information about polycystic ovarian syndrome**

a. Television

b. News paper

c. Friends

d. Health personnel

e. Books (magazine, journals)

f. Other sources (please mention) _________________

g. No previous information

….The end…

**Section B**

**Risk assessment questionnaire**

**(Polycystic ovarian syndrome)**

**------------------------------------------------------------------------------------------------------**

**Class and section: Date: Name of the school:**

**------------------------------------------------------------------------------------------------------**

**Instructions:** Please read all the questions carefully and indicate the response that you choose by placing a tick (√) in the space provided.

1. Height in cm---------2. Weight in Kg---------3. BMI--------

| **S. No** | **Questions** | **Never** | **Rarely** | **Some**  **time** | **Usually** |
| --- | --- | --- | --- | --- | --- |
| 1 | Do you have irregular periods ? |  |  |  |  |
| 2 | Do you miss your period in your regular cycle? |  |  |  |  |
| 3 | Do you get your menstrual cycle more than 35 days? |  |  |  |  |
| 4 | Do you have your menstrual flow for more than five days? |  |  |  |  |
| 5 | Are you changing more than 4 pads per day? |  |  |  |  |
| 6 | Do you experience Nausea/vomiting during menstruation? |  |  |  |  |
| 7 | Do you experience abdominal pain during menstruation? |  |  |  |  |
| 8 | Do you experience Pain/tenderness in breast during menstruation? |  |  |  |  |
| 9 | Do you have heavy menstrual flow with clots? |  |  |  |  |
| 10 | Do you have pimples on your face? |  |  |  |  |
| 11 | Do you have hair fall? |  |  |  |  |
| 12 | Do you have problem with hair growth over chest, face or abdomen |  |  |  |  |
| 13 | Do you have any darkening and thickening of skin folds around the neck and axillae |  |  |  |  |
| 14 | Do you have the habit of eating junk food? |  |  |  |  |
| 15 | Do you have weight gain? |  |  |  |  |
| 16 | Do you have difficulty in losing weight? |  |  |  |  |
| 17 | Do you have frequent thirst and urination? |  |  |  |  |
| 18 | Do you feel extremely hungry, irritable and sleepy? |  |  |  |  |
| 19 | Do you have symptoms of giddiness and fatigue |  |  |  |  |
| 20 | Do you have stressful and depressive mind? |  |  |  |  |
